# 1910. A Year's Work in the Hospitals and Medical Charities of London

**Published:** 1911-06-18

**Authors:** 


### 1910.

### A Year's Work in the Hospitals and Medical Charities of London.

### ST. MARYLEBONE AND WEST CENTRAL DISTRICT.

Comprising St. Marylebone, St. John's Wood, Bloomsbury, Holborn, etc.

| No. of Beds. | No. of Beds Daily Occupied. | Hospitals. | In-patients. | Out-patient Attendances. | Total Expenditure. | Income. Charitable. | Income. Proprietary. | Income. Patients' Payments. | Total Income. | Legacies not included in preceding column. |
|---|---|---|---|---|---|---|---|---|---|---|
| | | | | | £ | £ | £ | £ | £ | £ |
| 74 | 44 | French ... ... ... ... | 859 | 16,216 | 5,167 | 4,949 | 652 | ... | 5,601 | 1,187 |
| 50 | 48 | Italian ... ... ... ... | 903 | 9,278 | 3,089 | 2,701 | 359 | ... | 3,060 | ... |
| 104 | 68 | London Homœopathic ... ... | 831 | 49,907 | 10,617 | 3,136 | 3,829 | 1,157 | 8,122 | 4,955 |
| 110 | 99 | SS. John and Elizabeth ... ... | 540 | ... | 8,884 | 1,522 | 4,755 | 1,435 | 7,712 | ... |
| 413 | 390 | The Middlesex ... ... ... | 7,014 | 152,598 | 40,323 | 36,397 | 8,703 | ... | 45,10[illegible] | 15,829 |
| 100 | 98 | Alexandra, for Children... ... | 67 | 999 | 7,599 | 4,188 | 553 | 712 | 5,453 | 1,777 |
| 240 | 212 | Hospital for Sick Children ... | 3,297 | 99,489 | 22,552 | 10,141 | 5,295 | 210 | 15,646 | 9,554 |
| 24 | 22 | S. Monica's, for Children ... | 98 | ... | 1,323 | 748 | 253 | 275 | 1,276 | 250 |
| 28 | 19 | British Lying-In ... ... ... | 470 | 6,010 | 3,179 | 1,182 | 1,391 | 144 | 2,717 | ... |
| 82 | 72 | Queen Charlotte's Lying-In ... | 1,894 | 24,490 | 8,591 | 5,825 | 1,027 | 101 | 6,953 | 7,714 |
| 60 | 52 | New Hospital for Women ... | 770 | 30,753 | 8,675 | 2,903 | 1,166 | 1,676 | 5,745 | 1,428 |
| 56 | 52 | Samaritan Free ... ... ... | 935 | 15,883 | 5,339 | 4,116 | 499 | ... | 4,615 | 1,200 |
| 200 | 189 | National for the Paralysed, &c.... | 1,076 | 48,480 | 19,047 | 6,768 | 2,723 | 4,156 | 13,647 | 807 |
| 40 | 34 | Hospital for Epilepsy, &c. ... | 228 | 12,237 | 3,377 | 2,776 | 120 | 852 | 3,748 | 706 |
| 39 | 35 | West End, for Epilepsy, &c. ... | 237 | 25,618 | 7,070 | 4,524 | 271 | 343 | 5,138 | 11,625 |
| 26 | 19 | Central London Ophthalmic ... | 417 | 31,242 | 1,877 | 1,853 | 18 | ... | 1,871 | ... |
| 15 | 12 | Western Ophthalmic ... ... | 358 | 26,687 | 1,786 | 1,267 | 140 | 61 | 1,468 | 100 |
| 132 | 130 | Royal National Orthopædic ... | 767 | 19,501 | 12,981 | 7,587 | 821 | 1,064 | 9,472 | 853 |
| 31 | 20 | Hospital for Gentlewomen ... | 192 | ... | 11,932 | 1,526 | 153 | 1,373 | 3,052 | ... |
| ... | ... | National Dental ... ... ... | ... | 22,367 | 2,151 | 970 | ... | 1,416 | 2,386 | 225 |
| 14 | 11 | London Throat ... ... ... | 592 | 17,566 | 1,742 | 511 | ... | 1,232 | 1,743 | ... |
| 47 | 45 | The Middlesex Cancer ... ... | 153 | 524 | 4,767 | 2,582 | 2,317 | ... | 4,899 | 470 |
| 1,885 | 1,671 | | 21,698 | 609,845 | 192,068 | 108,172 | 35,045 | 16,207 | 159,424 | 58,680 |
| | | DISPENSARIES. | | | | | | | | |
| ... | ... | Bloomsbury Provident ... ... | ... | 5,147 | 242 | 73 | ... | 164 | 237 | ... |
| ... | ... | London Medical Mission ... | ... | 27,634 | 1,716 | 1,043 | 340 | 111 | 1,494 | 1,219 |
| ... | ... | Margaret Street, for Consumption | ... | 9,501 | 645 | 434 | 176 | ... | 610 | ... |
| ... | ... | St. John's Wood Provident ... | ... | 16,590 | 670 | 367 | 30 | 284 | 681 | 45 |
| ... | ... | St. Marylebone General ... ... | ... | 12,546 | 908 | 508 | 184 | 88 | 780 | ... |
| ... | ... | Western General ... ... ... | ... | 32,015 | 1,143 | 840 | 66 | 42 | 948 | 1,333 |
| 1,885 | 1,671 | | 21,698 | 713,278 | 197,392 | 111,437 | 35,841 | 16,896 | 164,174 | 61,277 |

### WESTMINSTER DISTRICT.—Comprising Westminster City and Liberties.

| No. of Beds. | No. of Beds Daily Occupied. | Hospitals. | In-patients. | Out-patient Attendances. | Total Expenditure. | Income. Charitable. | Income. Proprietary. | Income. Patients' Payments. | Total Income. | Legacies not included in preceding column. |
|---|---|---|---|---|---|---|---|---|---|---|
| | | | | | £ | £ | £ | £ | £ | £ |
| 237 | 139 | Charing Cross ... ... ... | 2,149 | 76,049 | 20,453 | 11,259 | 3,320 | 183 | 14,762 | 8,381 |
| 224 | 188 | King's College ... ... ... | 2,538 | 48,901 | 21,681 | 11,944 | 3,310 | 50 | 15,304 | 2,620 |
| 213 | 184 | Westminster ... ... ... ... | 2,610 | 74,938 | 20,315 | 7,282 | 4,353 | ... | 11,635 | 4,092 |
| 163 | 156 | Ventnor, for Consumption ... | 649 | ... | 13,562 | 5,309 | 2,750 | 4,420 | 12,479 | 2,042 |
| 36 | 31 | Grosvenor, for Women & Children | 417 | 9,254 | 2,855 | 1,520 | 357 | 754 | 2,631 | ... |
| 67 | 24 | Hospital for Women ... ... | 446 | 11,722 | 21,339 | 4,458 | 211 | 717 | 5,386 | 1,635 |
| 30 | 20 | Gordon, for Fistula ... ... | 338 | 5,484 | 3,092 | 453 | ... | 1,865 | 2,318 | ... |
| 26 | 24 | National, for Diseases of Heart... | 139 | 23,813 | 2,794 | 1,654 | 245 | 1,027 | 2,926 | 632 |
| 40 | 33 | Royal Westminster Ophthalmic... | 904 | 39,576 | 2,846 | 1,921 | 1,207 | ... | 3,128 | 20 |
| 20 | 13 | Royal Ear ... ... ... ... | 446 | 9,324 | 1,648 | 702 | 91 | 674 | 1,467 | 720 |
| ... | ... | Royal Dental ... ... ... | ... | 56,209 | 5,122 | 4,975 | 30 | 108 | 5,113 | 1,890 |
| 32 | 29 | St. Peter's, for Stone ... ... | 513 | 40,193 | 6,043 | 3,330 | 592 | 2,556 | 6,478 | 543 |
| 35 | 25 | St. John's, for Skin ... ... | 237 | 39,427 | 4,251 | 2,215 | 43 | 1,918 | 4,176 | ... |
| 85 | 68 | National Sanatorium, Bournem'th | 406 | ... | 3,647 | 2,621 | 241 | 1,247 | 4,109 | 105 |
| 50 | 42 | Infants Hospital, Vincent Square | 347 | 1,433 | 2,693 | 2,412 | 93 | ... | 2,505 | ... |
| 1,258 | 976 | | 12,139 | 436,323 | 132,341 | 62,055 | 16,843 | 15,519 | 94,417 | 22,680 |
| | | DISPENSARIES. | | | | | | | | |
| ... | ... | Public ... ... ... ... ... | ... | 9,867 | 725 | 298 | 242 | ... | 540 | ... |
| ... | ... | St. George's, Hanover Square ... | ... | 2,529 | 487 | 319 | ... | 150 | 469 | ... |
| ... | ... | Western ... ... ... ... | ... | 26,760 | 1,824 | 326 | 451 | 1,040 | 1,817 | ... |
| ... | ... | Westminster General ... ... | ... | 21,531 | 812 | 496 | 206 | 125 | 827 | ... |
| 1,258 | 976 | | 12,139 | 497,010 | 136,189 | 63,494 | 17,742 | 16,834 | 98,070 | 22,680 |

### CITY AND EAST CENTRAL DISTRICT.

Comprising the City, St. Luke's, Shoreditch, Finsbury, and Clerkenwell.

| No. of Beds. | No. of Beds Daily Occupied. | HOSPITALS. | In-patients. | Out-patient Attendances. | Total Expenditure. | Income. Charitable. | Income. Proprietary. | Income. Patients' Payments. | Total Income. | Legacies not included in preceding column. |
|---|---|---|---|---|---|---|---|---|---|---|
| | | | | | £ | £ | £ | £ | £ | £ |
| 680 | 567 | St. Bartholomew's ... ... | 7,870 | 350,580 | 81,821 | 5,015 | 68,257 | ... | 73,272 | 2,999 |
| 123 | 115 | Metropolitan ... ... ... | 1,656 | 153,396 | 18,944 | 14,059 | 685 | ... | 14,744 | 430 |
| 165 | 141 | Royal Free ... ... ... | 2,290 | 106,287 | 18,512 | 9,434 | 1,640 | ... | 11,074 | 30,303 |
| 80 | 67 | Royal, for Diseases of the Chest. | 690 | 25,540 | 6,920 | 5,888 | 216 | ... | 6,104 | 197 |
| 134 | 134 | Queen's, for Children ... ... | 1,970 | 84,317 | 14,404 | 11,921 | 416 | 581 | 12,918 | 805 |
| 50 | 33 | City of London Lying-In ... | 842 | 30,645 | 6,951 | 1,658 | 3,161 | 77 | 4,896 | 250 |
| 44 | 30 | St. Mark's, for Fistula ... ... | 430 | 7,487 | 6,953 | 3,987 | 563 | ... | 4,550 | 92 |
| 138 | 105 | Royal London Ophthalmic ... | 2,128 | 118,963 | 14,502 | 8,089 | 2,035 | ... | 10,124 | 9,229 |
| 26 | 22 | Central London Throat and Ear. | 726 | 53,665 | 3,601 | 1,285 | 232 | 3,114 | 4,631 | ... |
| 760 | 650 | | 10,732 | 580,300 | 90,787 | 56,321 | 8,948 | 3,772 | 69,041 | 41,306 |
| | | DISPENSARIES. | | | | | | | | |
| ... | ... | Billingsgate Medical Mission ... | ... | 15,454 | 678 | 567 | 92 | ... | 659 | ... |
| ... | ... | City ... ... ... ... | ... | 20,074 | 916 | 839 | 87 | ... | 926 | ... |
| ... | ... | Farringdon General ... ... | ... | 10,309 | 638 | 428 | 12 | 226 | 666 | ... |
| ... | ... | Finsbury ... ... ... ... | ... | 34,703 | 962 | 547 | 193 | 307 | 1,047 | 100 |
| ... | ... | Metropolitan ... ... ... | ... | 29,428 | 1,012 | 461 | 280 | 245 | 986 | ... |
| ... | ... | Royal General ... ... ... | ... | 11,254 | 815 | 291 | 394 | 94 | 779 | ... |
| 760 | 650 | | 10,732 | 701,522 | 95,808 | 59,454 | 10,006 | 4,644 | 74,104 | 41,406 |

### ISLINGTON AND NORTH-WEST DISTRICT.

Comprising Islington, Holloway, Highbury, Hampstead, Highgate, St. Pancras, Stoke Newington, Tottenham, &c.

| No. of Beds. | No. of Beds Daily Occupied. | HOSPITALS. | In-patients. | Out-patient Attendances. | Total Expenditure. | Income. Charitable. | Income. Proprietary. | Income. Patients' Payments. | Total Income. | Legacies not included in preceding column. |
|---|---|---|---|---|---|---|---|---|---|---|
| | | | | | £ | £ | £ | £ | £ | £ |
| 172 | 152 | Great Northern Central ... ... | 2,051 | 77,662 | 20,847 | 14,285 | 2,409 | 1,319 | 18,013 | 5,265 |
| 114 | 86 | Hampstead General Hospital ... | 1,315 | 46,250 | 10,674 | 7,216 | 290 | 913 | 8,419 | 3,133 |
| 100 | 84 | London Temperance ... ... | 1,286 | 71,729 | 10,405 | 4,935 | 1,880 | 303 | 7,118 | 4,475 |
| 125 | 107 | Tottenham (Prince of Wales's) .. | 1,638 | 86,316 | 9,231 | 9,606 | 124 | ... | 9,730 | 40 |
| 305 | 274 | University College ... ... | 4,189 | 146,585 | 27,369 | 20,845 | 3,909 | 58 | 24,812 | 9,802 |
| 230 | 214 | Mount Vernon for Consumption | 1,166 | 13,956 | 18,434 | 11,214 | 949 | ... | 12,163 | 6,979 |
| 14 | 13 | Children's Home Hospital, Barnet | 26 | ... | 1,564 | 1,413 | 85 | 53 | 1,551 | 500 |
| 189 | 41 | London Fever ... ... ... | 540 | ... | 9,271 | 6,236 | 1,541 | 2,314 | 10,091 | 2,890 |
| 25 | 16 | Invalid Asylum ... ... ... | 194 | ... | 909 | 364 | 411 | 125 | 900 | ... |
| 22 | 11 | Bushey Heath Cottage ... ... | 159 | ... | 696 | 379 | 126 | 228 | 733 | ... |
| 25 | 19 | Enfield Cottage ... ... ... | 317 | ... | 1,129 | 1,304 | 66 | 48 | 1,418 | ... |
| 27 | 15 | St. Saviour's Hospital ... ... | 166 | ... | 1,992 | 1,094 | 48 | 1,009 | 2,151 | ... |
| 48 | 40 | Friedenheim Hospital ... ... | 153 | ... | 3,652 | 2,488 | 284 | 333 | 3,105 | 3,509 |
| 25 | 20 | Willesden Cottage ... ... | 305 | 379 | 1,484 | 1,109 | 112 | 179 | 1,400 | 50 |
| 25 | 6 | Wood Green Cottage ... ... | 79 | ... | 1,835 | 609 | 42 | 128 | 779 | 2,404 |
| 20 | 19 | Santa Claus Home ... ... | 33 | ... | 880 | 719 | 46 | 67 | 832 | ... |
| 56 | 52 | Hospital for Incurable Children | 24 | ... | 2,056 | 1,217 | 386 | 777 | 2,380 | 65 |
| 18 | 17 | Winifred House, Holloway ... | 23 | ... | 837 | 532 | 38 | 156 | 726 | 385 |
| 16 | 15 | Highgate, All Saints' Home ... | 131 | ... | 361 | 406 | 3 | 3 | 412 | 90 |
| 18 | 14 | Erskine House, Hampstead ... | 188 | ... | 700 | 307 | ... | 393 | 700 | ... |
| 1,574 | 1,215 | | 13,983 | 442,877 | 124,254 | 86,278 | 12,749 | 8,406 | 107,433 | 39,587 |
| | | DISPENSARIES. | | | | | | | | |
| ... | ... | Camden Provident ... ... | ... | 6,056 | 285 | 62 | 45 | 168 | 275 | ... |
| ... | ... | Child's Hill, Provident ... ... | ... | 4,020 | 238 | 28 | 12 | 199 | 239 | ... |
| ... | ... | Hampstead Provident ... ... | ... | 25,327 | 980 | 261 | 58 | 672 | 991 | ... |
| ... | ... | Holloway and N. Islington ... | ... | 2,953 | 286 | 86 | 52 | 67 | 205 | ... |
| ... | ... | Islington ... ... ... ... | ... | 56,310 | 881 | 239 | 26 | 639 | 904 | 50 |
| ... | ... | Islington Medical Mission ... | ... | 4,522 | 461 | 426 | ... | 30 | 456 | ... |
| ... | ... | Kentish Town Medical Mission... | ... | 4,100 | 251 | 233 | ... | 8 | 241 | ... |
| ... | ... | St. Pancras and Northern ... | ... | 8,753 | 566 | 238 | 131 | 134 | 503 | ... |
| ... | ... | Stamford Hill, &c. ... ... | ... | 33,108 | 757 | 570 | 167 | ... | 737 | ... |
| 1,574 | 1,215 | | 13,983 | 588,026 | 128,959 | 88,421 | 13,240 | 10,323 | 111,984 | 39,637 |

### STRATFORD AND EAST-END DISTRICT.

Comprising Bethnal Green, Tower Hamlets, West Ham, Whitechapel, Hackney, Stepney, Limehouse, Poplar, and the East.

| No. of Beds. | No. of Beds Daily Occupied. | HOSPITALS. | In-patients. | Out-patient Attendances. | Total Expenditure. | Income. Charitable. | Income. Proprietary. | Income. Patients' Payments. | Total Income. | Legacies not included in preceding column. |
|---|---|---|---|---|---|---|---|---|---|---|
| | | | | | £ | £ | £ | £ | £ | £ |
| 130 | 117 | German ... ... ... ... | 1,704 | 89,001 | 12,831 | 7,375 | 3,682 | 725 | 11,782 | 10,735 |
| 922 | 803 | London ... ... ... ... | 14,507 | 598,467 | 119,103 | 58,669 | 34,281 | 2,403 | 95,353 | 24,738 |
| 50 | 42 | Mildmay Mission Hospital ... | 535 | 41,956 | 4,183 | 2,562 | 1,199 | 200 | 3,961 | 225 |
| 30 | 21 | Mildmay Memorial ... ... | 256 | ... | 1,790 | 483 | 1,082 | 178 | 1,743 | 50 |
| 103 | 80 | Poplar ... ... ... ... | 1,507 | 81,085 | 10,983 | 9,484 | 2,184 | 306 | 11,974 | 6,692 |
| 45 | 31 | Walthamstow, &c. ... ... | 554 | 13,349 | 1,650 | 1,453 | 141 | ... | 1,594 | ... |
| 60 | 48 | West Ham, &c. ... ... ... | 560 | 131,136 | 11,748 | 5,240 | 409 | ... | 5,649 | 500 |
| 170 | 152 | City of London for Dis. of the Chest | 952 | 40,140 | 12,317 | 12,739 | 533 | ... | 13,272 | 797 |
| 148 | 129 | East London for Children ... | 2,096 | 95,460 | 13,256 | 6,464 | 1,482 | ... | 7,946 | 6,986 |
| 35 | 33 | St. Mary's, Plaistow, for Children | 557 | 50,005 | 14,214 | 2,860 | 295 | 195 | 3,350 | 250 |
| 33 | 22 | East End Mothers' Home ... | 558 | 22,160 | 2,519 | 1,319 | 1,362 | 87 | 2,768 | ... |
| 23 | 21 | Canning Town Cottage ... ... | 313 | 17,252 | 1,709 | 858 | 508 | 359 | 1,725 | 75 |
| 14 | 10 | Passmore Edwards Cottage, T'lb'ry | 120 | 1,650 | 893 | 1,016 | 68 | ... | 1,084 | ... |
| 19 | 10 | East Ham Cottage ... ... | 154 | 15,558 | 912 | 970 | 98 | 90 | 1,158 | ... |
| 12 | 12 | Plaistow Maternity ... ... | 192 | ... | 743 | 438 | 92 | ... | 530 | ... |
| 1,794 | 1,531 | | 24,565 | 1,197,219 | 208,851 | 111,930 | 47,416 | 4,543 | 163,889 | 51,048 |
| | | DISPENSARIES. | | | | | | | | |
| ... | ... | All Saints, Buxton Street ... | ... | 3,159 | 157 | 111 | ... | ... | 111 | ... |
| ... | ... | Eastern ... ... ... ... | ... | 36,803 | 954 | 203 | 301 | 453 | 957 | 269 |
| ... | ... | Hackney Provident ... ... | ... | 1,879 | 378 | 129 | 4 | 242 | 375 | ... |
| ... | ... | London ... ... ... ... | ... | 6,030 | 462 | 113 | 269 | 3 | 385 | ... |
| ... | ... | Mildmay Medical Mission ... | ... | 4,799 | 135 | 36 | ... | 27 | 63 | ... |
| ... | ... | Queen Adelaide's ... ... ... | ... | 16,093 | 605 | 527 | 270 | ... | 797 | ... |
| ... | ... | Tower Hamlets ... ... ... | ... | 10,275 | 542 | 722 | 24 | 118 | 864 | ... |
| ... | ... | West Ham Provident ... ... | ... | 1,665 | 193 | 118 | 16 | 56 | 190 | ... |
| ... | ... | Whitechapel Provident ... ... | ... | 20,015 | 711 | 112 | ... | 593 | 705 | ... |
| 1,794 | 1,531 | | 24,565 | 1,297,937 | 212,988 | 114,001 | 48,300 | 6,035 | 168,336 | 51,317 |

### KENSINGTON AND WEST DISTRICT.

Comprising Kensington, Paddington, Bayswater, Kilburn, Chelsea, Brompton, Fulham, Hammersmith, Chiswick, Brentford, Acton, Ealing, etc.

| | | HOSPITALS. | | | £ | £ | £ | £ | £ | £ |
|---|---|---|---|---|---|---|---|---|---|---|
| 334 | 313 | St. George's ... ... ... | 4,857 | 122,894 | 40,223 | 9,671 | 14,599 | 141 | 24,411 | 16,146 |
| 301 | 275 | St. Mary's ... ... ... ... | 4,318 | 175,002 | 29,038 | 20,231 | 4,984 | 169 | 25,384 | 11,045 |
| 160 | 148 | West London ... ... ... | 2,413 | 151,214 | 15,594 | 12,914 | 838 | ... | 13,752 | 1,949 |
| 483 | 463 | Hospital for Consumption ... | 1,686 | 47,591 | 37,651 | 16,492 | 2,865 | 2,944 | 22,301 | 9,412 |
| 40 | 19 | Belgrave, for Children ... ... | 339 | 10,520 | 3,258 | 3,733 | 426 | 264 | 4,423 | 2,695 |
| 76 | 69 | Cheyne, for Sick & Incurable Chldn | 48 | ... | 3,693 | 1,309 | 1,201 | 451 | 2,961 | 744 |
| 14 | 6 | Kensington, General .. ... | 103 | 16,144 | 1,714 | 3,356 | 7 | 213 | 3,576 | ... |
| 13 | 9 | Kensington, for Children ... | 181 | 20,080 | 1,261 | 997 | 178 | ... | 1,175 | 250 |
| 46 | 36 | Paddington Green, for Children | 819 | 53,956 | 5,877 | 3,174 | 880 | 364 | 4,418 | 9,425 |
| 154 | 134 | Victoria, for Children ... ... | 1,703 | 69,599 | 10,960 | 7,046 | 868 | 416 | 8,330 | 2,632 |
| 50 | 47 | Chelsea, for Women ... ... | 868 | 10,197 | 14,312 | 3,625 | 364 | 1,025 | 5,014 | 10,453 |
| 104 | 85 | Cancer ... ... ... ... | 665 | 12,954 | 25,692 | 8,798 | 6,371 | ... | 15,169 | 12,842 |
| 145 | 114 | Female Lock ... ... ... | 312 | ... | 6,047 | 2,664 | 35 | 1,962 | 4,661 | 60 |
| 21 | 13 | Banstead Surgical Home ... | 84 | ... | 604 | 359 | 10 | 285 | 654 | 750 |
| 30 | 21 | Acton Cottage ... ... ... | 274 | 7,403 | 1,689 | 1,483 | 75 | 124 | 1,682 | 30 |
| 22 | 16 | Ealing Cottage ... ... ... | 223 | ... | 1,175 | 974 | 36 | 182 | 1,192 | ... |
| 16 | 9 | Epsom and Ewell Cottage ... | 139 | ... | 769 | 397 | 70 | 251 | 718 | ... |
| 16 | 11 | Hounslow Cottage ... ... | 142 | 3,115 | 743 | 477 | 175 | 60 | 712 | ... |
| 40 | 28 | Reigate and Redhill Cottage ... | 520 | ... | 1,954 | 1,291 | 87 | 282 | 1,660 | 200 |
| 24 | 21 | Teddington Cottage ... ... | 310 | ... | 1,068 | 1,047 | 99 | 47 | 1,193 | 70 |
| 18 | 16 | Wimbledon Cottage ... ... | 274 | 180 | 938 | 676 | 40 | 170 | 886 | ... |
| 14 | 10 | Wimbledon, South, Cottage ... | 264 | ... | 632 | 535 | ... | 140 | 675 | ... |
| 2,121 | 1,863 | | 20,542 | 700,849 | 204,892 | 101,249 | 34,208 | 9,490 | 144,947 | 78,703 |
| | | DISPENSARIES. | | | | | | | | |
| ... | ... | Brompton Provident ... ... | ... | 4,780 | 306 | 85 | ... | 192 | 277 | ... |
| ... | ... | Chelsea, &c. ... ... ... | ... | 5,455 | 561 | 263 | 65 | 43 | 371 | 7 |
| ... | ... | Chelsea Provident ... ... | ... | 1,524 | 260 | 58 | 18 | 183 | 259 | ... |
| ... | ... | Kensal Town Provident ... ... | ... | 3,829 | 267 | 37 | 34 | 188 | 259 | ... |
| ... | ... | Kilburn, Maida Vale ... ... | ... | 7,803 | 459 | 285 | 43 | ... | 328 | ... |
| ... | ... | Kilburn Provident ... ... | ... | 10,376 | 1,272 | 76 | 20 | 1,173 | 1,269 | ... |
| ... | ... | Notting Hill Provident ... ... | ... | 5,087 | 443 | 102 | 26 | 328 | 456 | ... |
| ... | ... | Paddington Provident ... ... | ... | 7,824 | 528 | 148 | 15 | 293 | 456 | ... |
| ... | ... | Royal Pimlico Provident... ... | ... | 19,015 | 673 | 368 | 97 | 180 | 645 | ... |
| ... | ... | Westbourne Provident ... ... | ... | 6,922 | 425 | 29 | 51 | 348 | 428 | ... |
| 2,121 | 1,863 | | 20,542 | 773,464 | 210,086 | 102,700 | 34,577 | 12,418 | 149,695 | 78,710 |

### NEWINGTON AND SOUTH DISTRICT.

Comprising Battersea, Wandsworth, Tooting, Balham, Streatham, Brixton, Lambeth, Newington, Southwark, Bermondsey, Camberwell, Greenwich, Deptford, Lewisham, Blackheath, Woolwich, etc.

| No. of Beds. | No. of Beds Daily Occupied. | HOSPITALS. | In-patients. | Out-patient Attendances. | Total Expenditure. | Income. | | | Total Income. | Legacies not included in preceding column. |
|---|---|---|---|---|---|---|---|---|---|---|
| | | | | | | Charitable. | Proprietary. | Patients' Payments. | | |
| | | | | | £ | £ | £ | £ | £ | £ |
| 617 | 533 | Guy's ... ... ... ... | 8,588 | 514,889 | 72,082 | 15,186 | 40,780 | 5,328 | 61,294 | 15,831 |
| 18 | 8 | Phillips' Memorial Homoeopathic | 138 | 2,312 | 1,210 | 612 | 226 | 275 | 1,113 | ... |
| 25 | 25 | Miller ... ... ... ... | 421 | 76,618 | 6,144 | 5,229 | 560 | 141 | 5,930 | 1,552 |
| 40 | 34 | St. John's, Lewisham ... ... | 301 | 1,390 | 2,646 | 1,952 | 93 | 440 | 2,485 | 145 |
| 604 | 516 | St. Thomas's ... ... ... | 7,316 | 84,287 | 71,279 | 3,249 | 59,567 | 494 | 63,310 | 3,680 |
| 306 | 258 | Seamen's ... ... ... ... | 2,371 | 122,251 | 23,032 | 16,964 | 2,768 | 870 | 20,602 | 1,373 |
| 76 | 57 | Evelina, for Children ... ... | 1,040 | 54,591 | 7,805 | 1,973 | 4,102 | 241 | 6,316 | 5,090 |
| 57 | 45 | Home for Sick Children... ... | 424 | 4,960 | 2,682 | 1,340 | 211 | 559 | 2,110 | 350 |
| 36 | 28 | General Lying-In... ... ... | 891 | 23,190 | 5,999 | 1,995 | 3,698 | ... | 5,693 | ... |
| 50 | 26 | Clapham Maternity & Dispensary | 619 | 12,802 | 1,836 | 517 | 700 | 725 | 1,942 | ... |
| 90 | 79 | Royal, Waterloo ... ... ... | 844 | 36,000 | 9,832 | 4,348 | 1,167 | ... | 5,515 | 254 |
| 42 | 31 | Royal Eye... ... ... ... | 728 | 75,470 | 6,063 | 3,383 | 530 | 912 | 4,825 | 538 |
| 28 | 15 | Beckenham Cottage ... ... | 246 | 789 | 1,294 | 1,023 | 19 | 373 | 1,415 | 500 |
| 20 | 15 | Blackheath Cottage ... ... | 161 | 6,470 | 1,176 | 945 | 41 | 197 | 1,183 | ... |
| 16 | 10 | Bromley Cottage... ... ... | 204 | 221 | 11,021 | 1,050 | 372 | 82 | 1,504 | 100 |
| 30 | 18 | Chislehurst, &c., Cottage ... | 306 | ... | 1,252 | 858 | ... | 510 | 1,368 | 50 |
| 22 | 12 | Eltham Cottage ... ... ... | 279 | ... | 1,137 | 775 | 155 | 205 | 1,135 | ... |
| 12 | 7 | Sidcup Cottage ... ... ... | 170 | 270 | 608 | 367 | 13 | 210 | 590 | ... |
| 30 | 15 | Livingstone Cottage ... ... | 199 | ... | 5,433 | 1,789 | 30 | 12 | 1,831 | ... |
| 60 | 50 | Bolingbroke Hospital ... ... | 814 | 32,044 | 6,136 | 3,026 | 288 | 764 | 4,078 | 1,300 |
| 24 | 16 | Victoria Hospital, Kingston ... | 221 | ... | 1,039 | 1,272 | 238 | 98 | 1,608 | ... |
| 13 | 8 | Woolwich Home for Mothers and Babies ... ... ... ... | 150 | 2,254 | 794 | 293 | 3 | 201 | 497 | ... |
| 2,216 | 1,806 | | 26,431 | 1,050,808 | 240,500 | 68,146 | 115,561 | 12,637 | 196,344 | 30,763 |
| | | DISPENSARIES. | | | | | | | | |
| ... | ... | Battersea Provident ... ... | ... | 204,000 | 5,091 | 195 | 61 | 4,866 | 5,122 | ... |
| ... | ... | Blackfriars, Provident ... ... | ... | 6,307 | 258 | 105 | ... | 154 | 259 | ... |
| ... | ... | Brixton, &c. ... ... ... | ... | 18,480 | 642 | 544 | 43 | 104 | 691 | ... |
| ... | ... | Camberwell Provident ... ... | ... | 91,170 | 1,536 | 246 | 193 | 1,061 | 1,500 | ... |
| ... | ... | Clapham ... ... ... ... | ... | 12,692 | 429 | 195 | 91 | 144 | 430 | 326 |
| ... | ... | Deptford Medical Mission ... | ... | 12,863 | 368 | 229 | 71 | 61 | 361 | ... |
| ... | ... | East Dulwich Provident ... | ... | 13,444 | 1,285 | 104 | 21 | 1,189 | 1,314 | 25 |
| ... | ... | Forest Hill Provident ... ... | ... | 5,475 | 571 | 175 | 16 | 374 | 565 | 125 |
| ... | ... | Greenwich Provident ... ... | ... | 25,381 | 573 | 55 | 10 | 500 | 565 | ... |
| ... | ... | Kennington, &c., Provident ... | ... | 4,993 | 218 | 108 | ... | 126 | 234 | ... |
| ... | ... | Royal South London ... ... | ... | 10,120 | 598 | 369 | 46 | ... | 415 | ... |
| ... | ... | South Lambeth, &c. ... ... | ... | 4,909 | 403 | 180 | 36 | 96 | 312 | ... |
| ... | ... | Walworth Provident ... ... | ... | 8,019 | 330 | 47 | 25 | 252 | 324 | ... |
| ... | ... | Wandsworth Common ... ... | ... | 2,897 | 250 | 21 | 11 | 218 | 250 | ... |
| ... | ... | Woolwich, &c., Provident ... | ... | 26,258 | 827 | 69 | 15 | 782 | 866 | ... |
| 2,216 | 1,806 | | 26,431 | 1,497,816 | 253,879 | 70,788 | 116,200 | 22,564 | 209,552 | 31,239 |

### THE MEDICAL CHARITIES OF LONDON.—A SUMMARY OF THE WORK DONE IN 1910.

It will be seen from the following summary that *One hundred and thirty thousand and ninety* patients were admitted into the Voluntary Hospitals and Medical Charities of London during the twelve months ending 31st December, 1910, and that the attendances in the Out-patient Departments and Dispensaries amounted to *Six millions sixty-nine thousand and fifty-three*, at a cost of *£1,235,301*. The Ordinary Income only amounted to *£975,915*, leaving a deficiency of *£259,386* on the year's work. The legacies received in 1910 amounted to *£326,266*.

| No. of Beds. | No. of Beds Daily Occupied. | HOSPITALS AND DISPENSARIES. | In-patients. | Out-patient Attendances. | Total Expenditure. | Income. | | | Total Income. | Legacies not included in preceding column. |
|---|---|---|---|---|---|---|---|---|---|---|
| | | | | | | Charitable. | Proprietary. | Patients' Payments. | | |
| | | | | | £ | £ | £ | £ | £ | £ |
| 2,216 | 1,806 | Newington and South District... | 26,431 | 1,497,816 | 253,879 | 70,788 | 116,200 | 22,564 | 209,552 | 31,239 |
| 760 | 650 | City and East Central District... | 10,732 | 701,522 | 95,808 | 59,454 | 10,006 | 4,644 | 74,104 | 41,406 |
| 1,258 | 976 | Westminster District ... ... | 12,139 | 497,010 | 136,189 | 63,494 | 17,742 | 16,834 | 98,070 | 22,680 |
| 1,885 | 1,671 | St. Marylebone and West Central District ... ... ... | 21,698 | 713,278 | 197,392 | 111,437 | 35,841 | 16,896 | 164,174 | 61,277 |
| 2,121 | 1,863 | Kensington and West District ... | 20,542 | 773,464 | 210,086 | 102,700 | 34,577 | 12,418 | 149,695 | 78,710 |
| 1,574 | 1,215 | Islington & North-West District | 13,983 | 588,026 | 128,959 | 88,421 | 13,240 | 10,323 | 111,984 | 39,637 |
| 1,794 | 1,531 | Stratford and East-End District | 24,565 | 1,297,937 | 212,988 | 114,001 | 48,300 | 6,035 | 168,336 | 51,317 |
| 11,608 | 9,712 | | 130,090 | 6,069,053 | 1,235,301 | 610,295 | 275,906 | 89,714 | 975,915 | 326,266 |

